# Sensitivity of Administrative Coding in Identifying Inpatient Acute Strokes Complicating Procedures or Other Diseases in UK Hospitals

**DOI:** 10.1161/JAHA.119.012995

**Published:** 2019-07-03

**Authors:** Linxin Li, Lucy E Binney, Samantha Carter, Sergei A. Gutnikov, Sally Beebe, Karen Bowsher‐Brown, Louise E. Silver, Peter M. Rothwell, the Oxford Vascular Study

**Affiliations:** ^1^ Centre for Prevention of Stroke and Dementia Nuffield Department of Clinical Neuroscience University of Oxford United Kingdom

**Keywords:** cerebrovascular disease/stroke, diagnostic coding, perioperative stroke, prospective cohort study, stroke, Epidemiology, Cerebrovascular Disease/Stroke

## Abstract

**Background:**

Administrative hospital diagnostic coding data are increasingly used in “big data” research and to assess complication rates after surgery or acute medical conditions. Acute stroke is a common complication of several procedures/conditions, such as carotid interventions, but data are lacking on the sensitivity of administrative coding in identifying acute stroke during inpatient stay.

**Methods and Results:**

Using all acute strokes ascertained in a population‐based cohort (2002–2017) as the reference, we determined the sensitivity of hospital administrative diagnostic codes (*International Classification of Diseases, Tenth Revision*;* ICD‐10*) for identifying acute strokes that occurred during hospital admission for other reasons, stratified by coding strategies, study periods, and stroke severity (National Institutes of Health Stroke Score</≥5). Of 3011 acute strokes, 198 (6.6%) occurred during hospital admissions for procedures/other diseases, including 122 (61.6%) major strokes. Using stroke‐specific codes (*ICD‐10*=I60–I61 and I63–I64) in the primary diagnostic position, 66 of the 198 cases were correctly identified (sensitivity for any stroke, 33.3%; 95% CI, 27.1–40.2; minor stroke, 30.3%; 95% CI, 21.0–41.5; major stroke, 35.2%; 95% CI, 27.2–44.2), with no improvement of sensitivity over time (*P*_trend_=0.54). Sensitivity was lower during admissions for surgery/procedures than for other acute medical admissions (n/% 17/23.3% versus 49/39.2%; *P*=0.02). Sensitivity improved to 60.6% (53.6–67.2) for all and 61.6% (50.0–72.1) for surgery/procedures if other diagnostic positions were used, and to 65.2% (58.2–71.5) and 68.5% (56.9–78.1) respectively if combined with use of all possible nonspecific stroke‐related codes (ie, adding *ICD‐10*=I62 and I65–I68).

**Conclusions:**

Low sensitivity of administrative coding in identifying acute strokes that occurred during admission does not support its use alone for audit of complication rates of procedures or hospitalization for other reasons.

**Clinical Perspective**

**What Is New?**

- Using 15‐year data from a population‐based stroke cohort with multiple overlapping ascertainment methods as the reference standard, we showed that administrative coding alone lacked sensitivity in identifying acute strokes that occurred during hospital stay for other diseases or as a complication of procedures.
- This poor sensitivity of coding also has not improved in the past 15 years.
- Depending on different code‐inclusion strategies, 40% to 70% of all strokes would have been missed if no additional ascertainment sources were used.

**What Are the Clinical Implications?**

- Studies that use hospital coding data alone could potentially underestimate complication rates.
- The lack of sensitivity does not support the use of administrative coding alone for assessing rates of acute stroke as complications.
- Approaches to improve coding accuracy for complications during acute admissions are required.

## Introduction

Routinely collected administrative hospital diagnostic coding data are inexpensive and widely available in electronic format and have long been used to audit complications of procedures, such as for carotid endarterectomy,^1^, ^2^, ^3^, ^4^ and are increasingly being used in “big data” research to assess complication rates following other surgery or acute medical conditions.^5^, ^6^, ^7^ In some countries, healthcare quality reporting is also derived from administrative data.^8^ However, validity of administrative data in identifying complications has varied in previous studies,^8^, ^9^, ^10^, ^11^, ^12^ with evidence of poor sensitivity,^9^, ^13^, ^14^ particularly for assessing safety outcomes after surgery.^7^, ^8^, ^9^, ^10^, ^11^, ^12^, ^15^, ^16^

Most previous studies evaluating coding sensitivity in assessing complication rates focused on the occurrence of infection or myocardial infarction during acute hospital admissions for other diseases or procedures,^9^, ^10^, ^12^, ^16^ but acute stroke is also a serious complication of several procedures or conditions.^17^ With increasing numbers of procedures being done in stroke prevention, such as carotid endarterectomy/stenting, catheter ablation for atrial fibrillation, or closure of patent foramen ovale, sensitivity of coding in ascertaining acute stroke complications during inpatient stay becomes increasingly important.^18^ Moreover, the primary diagnostic code (ie, the underlying cause) is commonly used in identifying hospital admissions following acute stroke,^19^ but the validity of this approach in identifying in‐hospital acute strokes is also unknown. Given that the primary diagnosis is usually considered as the condition that is mainly responsible for the admission to the hospital, using primary diagnosis alone may underestimate in‐hospital acute stroke cases, particularly during admissions for surgery or procedures. In the absence of similar studies, we aimed to use stroke cases ascertained in a population‐based cohort (Oxford Vascular Study) as the reference standard, to study the sensitivity of coding for identifying acute strokes during inpatient stay for procedures or other diseases. We also aimed to determine whether there was any improvement of sensitivity over time and compare approaches combining different stroke codes selection and diagnostic position.

## Methods

Requests for access to data from the Oxford Vascular Study will be considered by the corresponding author.

The Oxford Vascular Study (OXVASC) is an ongoing, population‐based study of the incidence and outcome of all acute vascular events. The study population comprises all 92 728 individuals, irrespective of age, registered with approximately 100 general practitioners in 9 general practices in Oxfordshire, United Kingdom.

The study methods have been reported elsewhere.^20^ Briefly, multiple overlapping methods of “hot” and “cold” pursuit were used to achieve near complete ascertainment of all individuals with transient ischemic attack or stroke. These include: (1) a daily, rapid access “transient ischemic attack and stroke clinic” to which participating general practitioners and the local emergency department refer individuals with suspected transient ischemic attack or minor stroke; (2) daily searches of admissions to the medical, stroke, neurology, and other relevant wards, including also screening all patients undergoing elective or emergency coronary, carotid, or peripheral vascular investigations or interventions; (3) daily searches of the local emergency department attendance register; (4) daily searches of in‐hospital death records through the bereavement office; (5) monthly searches of all death certificates and coroner's reports for out‐of‐hospital deaths; (6) monthly searches of all brain and vascular imaging referrals; and (7) monthly searches of general practitioner diagnostic coding and hospital electronic record discharge codes.

Patients with suspected stroke were seen by study physicians as soon as possible after the initial presentation. Stroke was defined as rapid‐onset symptoms and/or signs of focal, and at times global, loss of cerebral function, with symptoms lasting more than 24 hours or leading to death, with no apparent cause other than of vascular origin.^20^ Baseline demographic data, vascular risk factors, and other comorbidities were collected from face‐to‐face interview and cross‐referenced with primary care records. Detailed clinical history was recorded in all patients, and assessments were made for stroke severity using the National Institute of Health Stroke Scale. Major stroke was defined as National Institute of Health Stroke Scale≥5. For all acute strokes that occurred during inpatient stay for other diseases, we also recorded the reasons for the initial admission. Patients routinely had brain imaging, vascular imaging, 12‐lead ECG, and standard blood tests. If a patient died before assessment, we obtained an eyewitness account of the clinical event and reviewed any relevant records. All cases were reviewed by the senior study neurologist (P.M.R.) for final adjudication.

All patients were followed up face to face at 1, 6, 12, 60, and 120 months by a study nurse or physician to determine recurrent strokes. For patients who had moved out of the study area, telephone follow‐up was done. All patients were flagged for the Office for National Statistics mortality data, and all deaths during follow‐up were recorded with causes. All recurrent strokes that presented to medical attention would also be identified by the ongoing daily case ascertainment. If a recurrent stroke was suspected, the patient was reassessed and investigated by a study physician.

To assess the sensitivity of hospital diagnostic coding in identifying stroke cases, we used preselected *International Classification of Disease, Tenth Revision* (*ICD‐10*) codes (I60–I68; G45–G46; H34) that occurred at any diagnostic position. To assess the sensitivity of hospital diagnostic coding in identifying strokes occurring in‐hospital following procedures, all such cases were identified prospectively in OXVASC and cross‐referenced using the Office of Population, Censuses and Surveys: Classification of Interventions and Procedures, fourth Revision (OPCS‐4) classification to record details of surgical procedures performed.

### Statistical Analyses

Analysis was limited to acute strokes identified in OXVASC that happened during hospital admission for other diseases or procedures. To calculate sensitivity of hospital coding in identifying acute stroke episodes, we used all such strokes ascertained and adjudicated in OXVASC during 2002–2017 as the reference standard. We calculated sensitivity for each of 3 different coding inclusion strategies: (1) stroke‐specific codes (I60–I61, I63–I64) that appeared in the primary diagnostic position; (2) stroke‐specific codes (I60–I61, I63–I64) that appeared in any diagnostic position; and (3) all possible nonspecific stroke‐related codes (I60–I68) in any diagnostic position. These additional codes included I62 (subdural hemorrhage, nontraumatic extradural hemorrhage, and unspecified intracranial hemorrhage), I65 (occlusion and stenosis of precerebral arteries, not resulting in cerebral infarction), I66 (occlusion and stenosis of cerebral arteries, not resulting in cerebral infarction), I67 (other cerebrovascular diseases), and I68 (cerebrovascular disorders in diseases classified elsewhere).

Using the OXVASC data as the reference, we also compared the sensitivity of coding in identifying major versus minor strokes, ischemic versus hemorrhagic strokes, and admissions for surgery/procedures versus other medical admissions, using the chi‐square test. Time trends in coding sensitivity during the study period were assessed using the chi‐square test for trend. Analyses were stratified by different coding strategies and by reasons for initial admissions. Given the uncertainty of how previous stroke may affect the coders’ interpretation of the admission of interest, sensitivity analyses restricting to incident stroke cases were also performed.

We studied potential predictors for “false‐negative” coding by comparing the baseline characteristics of “true positive” versus the “false‐negative” cases using a *t* test for continuous variables and the chi‐square test for categorical variables. Univariate logistic regression was used to obtain odds ratios.

We did not have data of all nonstroke acute medical admissions or procedures in our study population and so could not determine the specificity of using coding in identifying acute strokes that happened during inpatient stay for procedures or other conditions.

All analyses were performed using SPSS software (version 22; SPSS, Inc, Chicago, IL).

### Standard Protocol Approval, Registration, and Patient Consent

Written informed consent or assent from relatives was obtained in all participants in OXVASC. OXVASC was approved by the local research ethics committee (OREC A: 05/Q1604/70).

## Results

Among a study population of 92 728, 3011 acute stroke episodes were ascertained in OXVASC, of which 236 (7.8%) occurred during inpatient stay, including 38 (16.1%) recurrent strokes that occurred during admissions of the index stroke. Of the remaining 198 acute stroke episodes that occurred during inpatient stay for other diseases (73 surgical/procedural and 125 acute medical admissions), 176 (88.9%) were ischemic strokes and 122 (61.6%) were major strokes (National Institute of Health Stroke Scale≥5).

Using stroke‐specific codes (I60–I61 and I63–I64) in primary diagnostic position, 66 acute strokes were correctly identified by coding (sensitivity, 30.3%; 95% CI, 21.0–41.5). Sensitivity improved to 60.6% (95% CI, 53.6–67.2) if other diagnostic positions were used and to 65.2% (95% CI, 58.2–71.5) if combined with all possible nonspecific stroke‐related codes (I60–I68). Similar patterns were found in analyses stratified by stroke subtypes, stroke severity, and for identifying acute strokes that occurred during both surgical and nonstroke medical admissions (Tables 1 and 2).

**Table 1:** Sensitivity of Hospital Diagnostic Coding in Identifying Acute Stroke During Hospital Admission for Other Reasons Stratified by Coding Strategies and Stroke Characteristics

	All	Subtypes			Severity*		
		Ischemic Stroke	Hemorrhagic Stroke	*P* Value	Major Stroke	Minor Stroke	*P* Value
	n (sensitivity, 95% CI)	n (sensitivity, 95% CI)	n (sensitivity, 95% CI)		n (sensitivity, 95% CI)	n (sensitivity, 95% CI)	
Incident and recurrent strokes	(n=198)	(n=176)	(n=22)		(n=122)	(n=76)	
Stroke‐specific codes in primary position	66 (33.3, 27.1–40.2)	53 (30.1, 23.8–37.3)	13 (59.1, 38.1–77.3)	0.007	43 (35.2, 27.2–44.2)	23 (30.3, 21.0–41.5)	0.47
Stroke‐specific codes in any position	120 (60.6, 53.6–67.2)	99 (56.3, 48.8–63.4)	21 (95.5, 73.6–99.4)	0.0004	78 (63.9, 55.0–72.0)	42 (55.3, 43.9–66.1)	0.23
Possible nonspecific stroke‐related codes in any position	129 (65.2, 58.2–71.5)	108 (61.4, 53.9–68.3)	21 (95.5, 73.6–99.4)	0.002	82 (67.2, 58.4–75.0)	47 (61.8, 50.4–72.1)	0.44
Incident strokes only	(n=153)	(n=135)	(n=18)		(n=89)	(n=64)	
Stroke‐specific codes in primary position	54 (35.3, 28.1–43.2)	44 (32.6, 25.2–41.0)	10 (55.6, 32.9–76.1)	0.06	33 (37.1, 27.6–47.6)	21 (32.8, 22.4–45.2)	0.59
Stroke‐specific codes in any position	97 (63.4, 55.4–70.7)	80 (59.3, 50.7–67.3)	17 (94.4, 69.0–99.2)	0.004	60 (67.4, 57.0–76.4)	37 (57.8, 45.4–69.3)	0.22
Possible nonspecific stroke‐related codes in any position	105 (68.6, 60.8–75.5)	88 (65.2, 56.7–72.8)	17 (94.4, 69.0–99.2)	0.01	63 (70.8, 60.5–79.3)	42 (65.6, 53.2–76.3)	0.50

Stroke‐specific codes included *International Classification of Diseases, Tenth Revision* (*ICD‐10*) codes: I60, I61, I63, and I64; all possible nonspecific stroke‐related codes included *ICD‐10* codes: I60‐I68.

* Severity is defined by the National Institutes of Health Stroke Score (NISSS): major stroke (NIHSS≥5) and minor stroke (NIHSS<5).

**Table 2:** Sensitivity of Hospital Diagnostic Coding in Identifying the Occurrence of Inpatient‐Stroke Episodes Stratified By Coding Stratifies and the Initial Reasons for Hospital Admission

	After Procedures/Surgery*	After Other Admissions	*P* Value
	n (sensitivity, 95% CI)	n (sensitivity, 95% CI)	
Incident and recurrent strokes	(n=73)	(n=125)	
Stroke‐specific codes in primary position	17 (23.3, 15.0–34.4)	49 (39.2, 31.0–48.1)	0.02
Stroke‐specific codes in any position	45 (61.6, 50.0–72.1)	75 (60.0, 51.1–68.3)	0.82
Possible nonspecific stroke‐related codes in any position	50 (68.5, 56.9–78.1)	79 (63.2, 54.4–71.2)	0.45
Incident strokes only	(n=57)	(n=96)	
Stroke‐specific codes in primary position	10 (17.5, 9.7–29.7)	44 (45.8, 36.1–55.9)	0.0004
Stroke‐specific codes in any position	35 (61.4, 48.2–73.1)	62 (64.6, 54.5–73.5)	0.69
Possible nonspecific stroke‐related codes in any position	40 (70.2, 57.0–80.7)	65 (67.7, 57.7–76.3)	0.75

Stroke‐specific codes included *International Classification of Diseases, Tenth Revision* (*ICD‐10*) codes: I60, I61, I63, and I64; all possible nonspecific stroke‐related codes included *ICD‐10* codes: I60 to I68.

* n=24 postcardiothoracic; n=22 postorthopedic; n=5 after carotid endarterectomy/stent; n=22 after other surgical procedures.

Coding sensitivity did not differ by stroke severity and was similarly low for identifying minor versus major strokes (Table 1). However, coding had significantly lower sensitivity in identifying ischemic than hemorrhagic strokes (stroke specific codes in any position—ischemic stroke 56.3% versus 95.5%; *P*=0.0004; Table 1). There was no trend of improvement during the study period (Table 3).

**Table 3:** Temporal Trends of Sensitivity of Hospital Diagnostic Coding in Identifying the Occurrence of Acute Stroke During Hospital Admission, Stratified by Coding Strategies, Stroke Subtypes, and the Initial Reasons for Admission

Subgroups by Coding Strategies	No. (%) of Correctly Identified Cases by Coding			
	2002–2007	2007–2012	2012–2017	*P*_trend_
Overall				
All incident and recurrent strokes	(n=68)	(n=66)	(n=64)	
Stroke‐specific codes in primary position	20 (29.4)	24 (36.4)	22 (34.4)	0.54
Stroke‐specific codes in any position	43 (63.2)	36 (54.5)	41 (64.1)	0.94
Possible nonspecific stroke‐related codes in any position	44 (64.7)	41 (62.1)	44 (68.8)	0.64
Incident strokes only	(n=54)	(n=48)	(n=51)	
Stroke‐specific codes in primary position	17 (31.5)	16 (33.3)	21 (41.2)	0.30
Stroke‐specific codes in any position	36 (66.7)	25 (52.1)	36 (70.6)	0.70
Possible nonspecific stroke‐related codes in any position	37 (68.5)	30 (62.5)	38 (74.5)	0.52
By stroke subtypes				
Ischemic strokes	(n=59)	(n=61)	(n=56)	
Stroke‐specific codes in primary position	16 (27.1)	20 (32.8)	17 (30.4)	0.70
Stroke‐specific codes in any position	34 (57.6)	31 (50.8)	34 (60.7)	0.75
Possible nonspecific stroke‐related codes in any position	35 (59.3)	36 (59.0)	37 (66.1)	0.46
Hemorrhagic strokes	(n=9)	(n=5)	(n=8)	
Stroke‐specific codes in primary position	4 (44.4)	4 (80.0)	5 (62.5)	0.44
Stroke‐specific codes in any position	9 (100.0)	5 (100.0)	7 (87.5)	0.23
Possible nonspecific stroke‐related codes in any position	9 (100.0)	5 (100.0)	7 (87.5)	0.23
By initial reasons for admission				
Inpatient stroke after procedures/surgery*	(n=27)	(n=20)	(n=26)	
Stroke‐specific codes in primary position	5 (18.5)	8 (40.0)	4 (15.4)	0.80
Stroke‐specific codes in any position	18 (66.7)	11 (55.0)	16 (61.5)	0.70
Possible nonspecific stroke‐related codes in any position	19 (70.4)	13 (65.0)	18 (69.2)	0.93
Inpatient stroke after acute medical admissions	(n=41)	(n=46)	(n=38)	
Stroke‐specific codes in primary position	15 (36.6)	16 (34.8)	18 (47.7)	0.34
Stroke‐specific codes in any position	25 (61.0)	25 (54.3)	25 (65.8)	0.68
Possible nonspecific stroke‐related codes in any position	25 (61.0)	28 (60.9)	26 (68.4)	0.83

Stroke specific codes included *International Classification of Diseases, Tenth Revision* (*ICD‐10*) codes: I60, I61, I63, and I64; all possible nonspecific stroke‐related codes included *ICD‐10* codes: I60 to I68.

* n=24 post cardiothoracic; n=22 postorthopaedic; n=5 after carotid endarterectomy/stent; n=22 after other surgical procedures.

Among the 73 strokes that occurred during admissions for surgery or other procedures, 24 were postcardiothoracic surgery, 22 postorthopedic surgery, 5 after carotid stenting or carotid endarterectomy, and 22 were after other types of surgical procedures. Sensitivity of coding to identify such strokes was lower after surgery than after other acute medical admissions if stroke‐specific codes in primary position were used (23.3% versus 39.2%; *P*=0.02; Table 2). However, this difference disappeared if other diagnostic positions were included or if all stroke‐related codes in any position were used (Table 2). Results were also consistent if only incident stroke cases were included (Table 2). Again, no temporal trend of improvement of sensitivity was observed during the study period (Table 3).

We also attempted to study predictors of “false‐negative” coding of acute strokes that occurred during hospital stay. In addition to the differences in relation to ischemic versus hemorrhagic strokes, not being transferred to the acute stroke unit was also positively associated with “false‐negative cases” (stroke‐specific codes in primary position: odds ratios=3.7; 95% CI, 1.9–7.4; *P*=0.0001; stroke‐specific codes in any position: odds ratios=4.5; 95% CI, 2.3–8.8; *P*<0.0001; Table 4), and nontransfer to stroke unit was justified in most cases by complexities of specialist care, such as postsurgical management. However, there was no significant difference in age, sex, distribution of vascular risk factors, length of hospital stay, or days from the acute stroke to the initial admission between the “true‐positive” and “false‐negative” cases (Table 4).

**Table 4:** Baseline Characteristics of “True‐Positive” vs “False‐Negative” Cases

	Total	I60, I61, I63, and I64 in Primary Position			I60, I61, I63, and I64 in Any Position		
		True Positive	False Negative	*P* Value	True Positive	False Negative	*P* Value
	(n=198)	(n=66)	(n=132)		(n=120)	(n=78)	
Demographics							
Age, y (mean/SD)	77.3/11.0	78.9/10.4	76.5/11.2	0.16	77.0/11.6	77.8/9.9	0.64
Male sex	97 (49.0)	28 (42.4)	69 (52.3)	0.19	58 (48.3)	39 (50.0)	0.82
Stroke subtypes							
Ischemic stroke	176 (88.9)	53 (80.3)	123 (93.2)	0.007	99 (82.5)	77 (98.7)	0.0004
Hemorrhagic stroke*	22 (11.1)	13 (19.7)	9 (6.8)		21 (17.5)	1 (1.3)	
Stroke severity							
Major stroke (NIHSS≥5)	122 (61.6)	42 (65.2)	79 (59.8)	0.47	78 (65.0)	44 (56.4)	0.23
Minor stroke (NIHSS<5)	76 (38.4)	23 (34.8)	53 (40.2)		42 (35.0)	34 (43.6)	
Previous medical history							
Stroke	45 (22.7)	12 (18.2)	33 (25.0)	0.28	23 (19.2)	22 (28.2)	0.14
Myocardial infarction	39 (19.7)	10 (15.2)	29 (22.0)	0.67	24 (20.0)	15 (19.2)	0.89
PVD	30 (15.2)	9 (13.6)	21 (15.9)	0.26	17 (14.2)	13 (16.7)	0.63
Hypertension	125 (63.1)	47 (71.2)	78 (59.1)	0.10	79 (65.8)	46 (59.0)	0.33
Diabetes mellitus	39 (19.7)	12 (18.2)	27 (20.5)	0.71	25 (20.8)	14 (17.9)	0.62
Hyperlipidemia	60 (30.3)	18 (27.3)	42 (31.8)	0.51	34 (28.3)	26 (33.3)	0.45
Atrial fibrillation	90 (45.5)	34 (51.5)	56 (42.4)	0.23	57 (47.5)	33 (42.3)	0.47
Valvular heart disease	37 (18.7)	16 (24.2)	21 (15.9)	0.16	25 (20.8)	12 (15.4)	0.34
Cardiac failure	40 (20.2)	15 (22.7)	25 (18.9)	0.53	21 (17.5)	19 (24.4)	0.24
Current smoker†	21 (10.6)	6 (9.4)	15 (11.8)	0.61	10 (8.7)	11 (14.5)	0.21
Characteristics of the admission							
Length of hospital stay (median/IQR)	17 (8–33)	15 (6–29)	19 (8–35)	0.29	18 (9–34)	16 (17–32)	0.66
Days postadmission (median/IQR)	3 (1–9)	2 (0–6)	4 (1–9)	0.06	3 (1–9)	4 (1–8)	0.21
Admission to ASU	73 (36.9)‡	36 (65.5)‡	37 (33.6)‡	0.0001	56 (58.9)‡	17 (24.3)‡	<0.0001

Data are presented as n (%), unless specified. ASU indicates acute stroke unit; IQR, interquartile range; NIHSS, National Institutes of Health Stroke Score; PVD, peripheral vascular disease.

* Including intracerebral hemorrhage and subarachnoid hemorrhage.

† Data missing for n=7.

‡ ASU was only opened in the catchment area from 2005, and 33 stroke cases that happened before 2005 were not included.

## Discussion

Using a population‐based stroke cohort with multiple overlapping ascertainment methods as the reference standard, we showed that, in the United Kingdom at least, administrative hospital coding alone lacked sensitivity in identifying acute strokes that occurred during hospital stay for other diseases or as a complication of procedures. This poor sensitivity of coding also has not improved over time. Depending on different code‐inclusion strategies, 40% to 70% of all strokes would have been missed if no additional ascertainment sources were used. Consequently, studies that use hospital coding data alone would significantly underestimate complication rates.

The low sensitivity of coding we found for acute stroke was similar to the estimates for myocardial infarction reported by 2 previous studies.^9^, ^12^ Maass et al reported that the coding sensitivity for identifying myocardial infarction as a complication was 20.8% in a German cohort of acute admissions.^9^ Parthasarathy et al also found that hospital coding was poor to moderate for ascertaining periprocedure myocardial infarction.^12^ The poor sensitivity of coding in identifying acute stroke that occurred during inpatient stay for other diseases is perhaps not surprising. In many countries, including the United Kingdom, hospital diagnostic coding is often done by nonclinical clerical staff and largely depends on their interpreting of medical notes and applying appropriate codes. The actual reason of the acute admission is not always clear in retrospect, and complications may get missed in patients with multiple comorbidities. Moreover, there might be inadequate documentation in the medical records, leading to subsequent coding errors.

The poor sensitivity of coding in identifying acute strokes during hospital admissions for procedures or other diseases was also supported by previous studies addressing the unreliability of administrative coding data for determining perioperative stroke after carotid endarterectomy or carotid stenting.^15^, ^18^ Bensley et al found that the sensitivity of coding data for determining perioperative stroke was 66.7%, which was also consistent with our estimates.^15^ Systematic review of cohort studies of carotid endarterectomy for symptomatic stenosis showed that the proportions of nonfatal operative strokes in surgeon‐only studies were lower than that reported in studies that involved neurologists for adjudication of outcomes,^21^ suggesting that “false‐negative” coding cases may be partly related to under‐reporting leading to perioperative acute stroke cases being missed by coders subsequently.

We found that coding sensitivity increased if the nonprimary diagnostic positions were used. Although this approach is at the expense of a lower specificity and positive predictive value because pre‐existing conditions may be inappropriately coded,^19^, ^22^ it is perhaps a better strategy in this setting given that the initial reasons for the acute admissions are perhaps intuitively more likely to be chosen as the primary diagnosis. We also showed that the combination of using all stroke‐related codes (I60–I68) and nonprimary diagnostic position increased sensitivity further, albeit only by a small amount. Given that using all stroke‐related codes would further decrease positive predictive value and specificity, and some of the codes are strongly associated with the procedures of interest, for example I65.2 occlusion and stenosis of carotid artery is strongly associated with carotid stenting or carotid endarterectomy, using all possible nonspecific stroke‐related codes might overestimate risks of acute stroke during carotid procedures.

Our study findings do not support routine use of coding data alone in assessing perioperative acute stroke rates or in monitoring acute stroke as a complication during inpatient stay for other nonstroke conditions. If no additional ascertainment sources were used, up to 70% of the true cases could have been missed. More important, underestimation of acute stroke as a complication in a nontrial population may provide false reassurance of generalizability of safety profiles of a procedure demonstrated in randomized trials.

We did not find any clinical predictors for “false‐negative” coding cases. However, “false‐negative” cases were more frequently observed for ischemic than for hemorrhagic strokes. Therefore, any underascertainment of acute strokes by hospital coding is likely driven by underestimation of ischemic strokes. This would have implications in studies addressing risk and benefit of a procedure in stroke prevention, with potential overestimation of benefit (eg, preventing ischemic stroke versus causing hemorrhagic stroke).

Although we consider our results to be valid, our study has some limitations. First, our study was done in Oxfordshire and might not be representative of all hospitals in the United Kingdom. However, our estimates were highly comparable to other validation studies in the United Kingdom.^12^ Second, given that coding accuracy might differ between healthcare systems, the coding sensitivity we found might not be generalizable to other countries, especially in countries where accurate coding is linked to additional hospital income. Nevertheless, validity of coding in identifying complications during hospital stay for other diseases has also been questioned in the United States, Canada, and other European countries.^8^, ^9^, ^11^, ^13^ Third, our statistical power is limited, especially for the analyses looking at predictors for “false‐negative” cases, and we are not powered to reliably test whether a delay from admission to onset of acute stroke was associated with stroke diagnoses missed by coding. Fourth, with increasing physician input on surgical wards, only one‐third of the acute strokes that happened during inpatient stay for procedures or other diseases were transferred to the stroke unit in our study. Therefore, diagnostic uncertainty from the attending teams could be a reason for acute stroke cases being missed by coders. However, we did not systematically compare the medical notes and could not reliably tell how many of the missed stroke cases were attributed to diagnosis uncertainty. Finally, we did not have data of all nonstroke acute medical admissions or procedures in our study population and therefore could not determine the specificity of using coding in identifying acute strokes that happened during inpatient stay for other conditions.

In conclusion, we showed poor sensitivity of hospital diagnostic coding in identifying acute stroke cases that occurred during inpatient stay for other diseases, with no improvement in the past 15 years in Oxfordshire, United Kingdom. Although we could not determine specificity of administrative coding in identifying acute strokes that occurred during admission for procedures or other diseases, the lack of sensitivity does not support its use alone for assessing rates of acute stroke as complications. Approaches to improve coding accuracy for complications during acute admissions are required.

## Author Contributions

Linxin Li collected data, did the statistical analysis and interpretation, and wrote and revised the manuscript. Lucy Binney, Samantha Carter, Sergei Gutnikov, Sally Beebe, Karen Bowsher‐Brown, and Louise Silver collected data. Peter Rothwell conceived and designed the overall study, provided study supervision and funding, acquired, analyzed, and interpreted data, and wrote and revised the manuscript.

## Source of Funding

The Oxford Vascular Study is funded by the National Institute for Health Research (NIHR) Oxford Biomedical Research Centre (BRC), Wellcome Trust, Wolfson Foundation, and British Heart Foundation. Rothwell is in receipt of an NIHR Senior Investigator award. The views expressed are those of the author(s) and not necessarily those of the National Health Service (NHS), the NIHR, or the Department of Health.

## Disclosures

None.

## Supporting information


**Appendix S1.** Oxford Vascular Study Group Members (current research staff).
