# Supplementary material for: Sensitivity of Administrative Coding in Identifying Inpatient Acute Strokes Complicating Procedures or Other Diseases in UK Hospitals
Source: J Am Heart Assoc. 2019 Jul 3;8(14):e012995. doi: 10.1161/JAHA.119.012995 (PMC6662118; doi:10.1161/JAHA.119.012995)
Supplement: Supplementary file 1 — Appendix S1. Oxford Vascular Study Group Members (current research staff). [file JAH3-8-e012995-s001.pdf]

SUPPLEMENTAL MATERIAL

Appendix

Oxford Vascular Study group members (current research staff)

**Name**	**Role**	**Institution**
Prof Peter M Rothwell	Director	University of Oxford
Dr Louise Silver	Research Coordinator	University of Oxford
Prof Sarah Pendlebury	Senior Research Fellow	University of Oxford
Dr Wilhelm Kuker	Neuro-radiologist	University of Oxford
Dr Alastair Webb	Senior Research Fellow	University of Oxford
Dr Linxin Li	Senior Research Fellow	University of Oxford
Dr Sara Mazzucco	Senior Research Fellow	University of Oxford
Dr Gabriel Yiin	Senior Research Fellow	University of Oxford
Dr Maria Tuna	Senior Research Fellow	University of Oxford
Dr Ramon Luengo-Fernandez	Senior Research Fellow	University of Oxford
Dr Lucy Binney	Senior Research Fellow	University of Oxford
Dr Ziyah Mehta	Senior Statistician	University of Oxford
Dr Sergei Gutnikov	Database Manager	University of Oxford
Ms Jean Brooks	Research Secretary	University of Oxford
Dr Aubretia McColl	Clinical Research Fellow	University of Oxford
Dr Iain McGurgan	Clinical Research Fellow	University of Oxford
Dr Robert Hurford	Clinical Research Fellow	University of Oxford
Dr Dearbhla Kelly	Clinical Research Fellow	University of Oxford
Ms Sarah Welch	Research Nurse	University of Oxford
Ms Michelle Wilson	Research physiotherapist	University of Oxford
Ms Fiona Cuthbertson	Research physiotherapist	University of Oxford
Ms Ellen McCulloch	Research Nurse	University of Oxford
Ms Sally Beebe	Research Nurse	University of Oxford
Ms Karen Bowsher-Brown	Research Nurse	University of Oxford
Ms Josephine Brooks	Research Nurse	University of Oxford
Ms Susannah Rae	Research Nurse	University of Oxford
Ms Emily-Rose Vaughan-Fowler	Research Nurse	University of Oxford
Ms Robyn Harris	Research Nurse	University of Oxford
Ms Anne-Marie Haigh	Research Nurse	University of Oxford
Ms Amy Lawson	Research Assistant	University of Oxford
Dr Annette Burgess	Laboratory Manager	University of Oxford
Ms Deborah Poole	Laboratory Technician	University of Oxford
Ms Julia Duerden	Laboratory Technician	University of Oxford
